# Ecological Transition and Sustainable Development, A Multivariate Statistical Analysis to Guide the Policies of the National Recovery and Resilience Plan

**DOI:** 10.1007/s11205-023-03078-w

**Published:** 2023-02-03

**Authors:** Paola Perchinunno, Antonella Massari, Samuela L’Abbate, Lucia Mongelli

**Affiliations:** 1 Department of Economics, Management and Business Law, University of Bari “Aldo Moro”, Largo Abbazia Santa Scolastica 53, 70124 Bari, Italy; 2 ISTAT - Dipartimento Per La Produzione Statistica, Direzione Centrale Della Raccolta Dati - Servizio per la Raccolta Dati per le Statistiche Economiche e Ambientali, Piazza A. Moro, 61, 70122 Bari, Italy

**Keywords:** Statistical indicators, Socio-economic development, Ecological transition, Sustainable development goals, Fuzzy logic, DBSCAN

## Abstract

The sustainable development goals are a call to action by all upper-middle-income countries and developing countries to promote development while protecting the planet. The United Nations defines sustainable "a development that meets the needs of the present without compromising the ability of future generations to meet their own needs". The present work proposes a first mapping of the correspondences between the SDGs and the 6 Missions envisaged by the National Recovery and Resilience Plan (NRRP). In this paper, the data referred to Mission 2 (Green Revolution and Ecological Transition) will be analyzed at a provincial level in order to evaluate the territorial adequacy of the economic planning through multivariate statistical methodologies (Totally Fuzzy and Relative method and DBScan). Data analysis allows to develop an integrated approach for the evaluation of the government policies of the territory and for monitor the progress of the subsequent intervention policies of the Italian Government.

## Introduction

The Sustainable Development Goals are a call to action by all upper-middle-income and developing countries to promote development while protecting the planet. The UN defines sustainable "a development that meets the needs of the present without compromising the ability of future generations to meet their own needs". A complex and articulated concept of responsibilities that concern all countries and all peoples called to contribute to the effort to bring the world on a sustainable path, without distinction between developed, emerging and developing countries.

At the European level, the SDGs have already entered among the tools used to monitor the development of a country. With respect to this scenario, the National Recovery and Resilience Plan (NRRP) illustrates the areas of intervention through some indicators in the social, economic, and environmental fields (Gibson, 2006; Kumar et. Al, 2012). Interesting in the scientific debate is the use of sustainability indicators (SDGs) for monitoring the NRRP.

The present work proposes a first mapping of the correspondences between the SDGs and the 6 Missions envisaged by the Plan with reference to Mission 2 (Green Revolution and Ecological Transition) and therefore to the statistical indicators connected to it. Specifically, the issue of ecological transition with the connected statistical indicators is of great interest.

The ecological transition is the process of technological innovation to achieve a change in our society considering compliance with the criteria for environmental sustainability. The term "ecological transition" appears for the first time during the 1970s, especially in the 1972 Meadows report, which insists on the need for a "transition from a growth model to one of global equilibrium", underlining the ecological risks induced by economic and demographic growth. In 1987, the Brundtland report (Boissonade, 2017) recommended "the transition to sustainable development".

The presence of multiple data allows to develop an integrated approach to the evaluation of the government policies of the territory in place (Cohen, 2017; Bond, 2011; Fitzgerald et al., 2012) and to be able to monitor the progress of the subsequent intervention policies of the Italian government. It has already been affirmed by other authors (Prota & Viesti, 2022) the need to evaluate the effects of the NPPR on the other policies in progress in the European states in the period 2021–2027, demonstrating the importance of this evaluation for the successful outcome of the expenditure both from an economic point of view and under the environmental profile. In this paper, the data referred to Mission 2 (Green Revolution and Ecological Transition) will be analyzed at a provincial level to evaluate the territorial adequacy of the economic planning through multivariate statistical methodologies (Totally Fuzzy and Relative method and DBScan).

## The Statistical Indicators of the Sdgs Report (Sustainable Development Goals)

### The Project SDGs

On 25 September 2015, the United Nations General Assembly adopted the 2030 Agenda for Sustainable Development which sets out the global objectives to end poverty, protect the planet and ensure prosperity for all by 2030: the Sustainable.

Development Goals (SDGs). Each country must commit to defining its own sustainable development strategy that will enable it to achieve the objectives of the 2030 Agenda.

With the adoption of the 2030 Agenda, countries have voluntarily submitted to the monitoring process carried out directly by the United Nations with respect to the state of implementation of the SDGs.

Istat, like the other National Statistical Institutes, is called by the United Nations Statistical Commission to play a role of national coordination in the production of indicators for the measurement of sustainable development and the monitoring of its objectives.

The 17 "Sustainable Development Goals (SDGs)" and their 169 targets refer to a set of issues important for development and outline global action plan for the coming years.

**Fig. 1:**
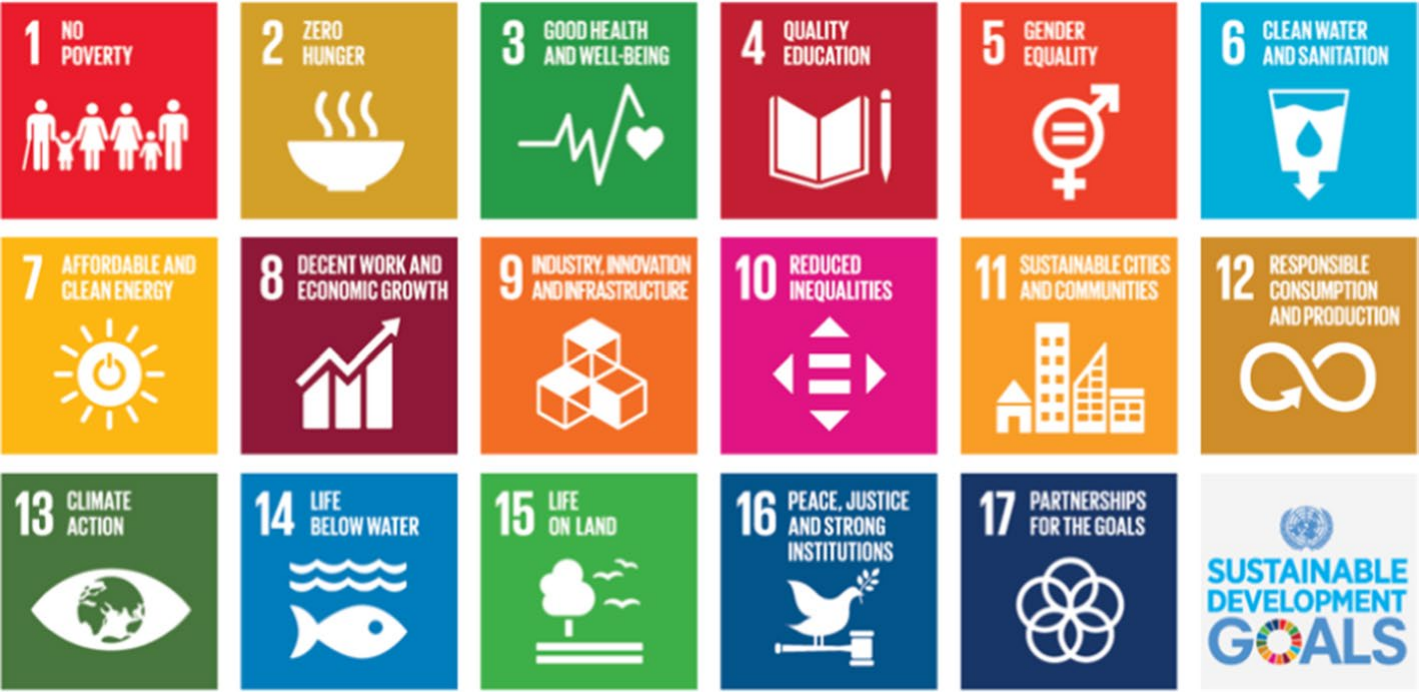
Sustainable Development Goals Agenda 2030

Below is a brief development of the 17 objectives (Fig. 1):

1. Ending all forms of poverty in the world
2. Ending hunger, achieving food security, improving nutrition and promoting sustainable agriculture
3. Ensuring health and well-being for all ages
4. Provide quality, equitable and inclusive education and learning opportunities for all
5. Achieving gender equality and empowering all women and girls
6. Ensure the availability and sustainable management of water and sanitation for all
7. Ensure access to affordable, reliable, sustainable and modern energy systems for all
8. Fostering lasting, inclusive and sustainable economic growth, full and productive employment and decent work for all
9. Build a resilient infrastructure and promote innovation and fair, responsible and sustainable industrialization
10. Reducing inequality within and between nations
11. Making cities and human settlements inclusive, safe, durable and sustainable
12. Ensuring sustainable production and consumption patterns
13. Promote action, at all levels, to combat climate change
14. Conserve and sustainably use the oceans, seas and marine resources for sustainable development
15. Protect, restore, and promote a sustainable use of the earth's ecosystem
16. Promoting peaceful and inclusive societies for sustainable development
17. Strengthening the means of implementation and renewing the Global Partnership for Sustainable Development.

Every year Istat publishes the Report on the SDGs (ISTAT, 2021); the fourth report of 22 August 2021 offers a first representation of the impact of the pandemic on SDGs indicators. The fourth Report was last updated on February 18, 2022.

The update summarizes: 367 statistical measures (of which 338 are different) 138 indicators United Nations Inter Agency Expert Group (UN-IAEG), which constitute the global reference framework with the usual regional analysis, useful for the observation of territorial imbalances. It is a system of indicators of great complexity that sees within it both consolidated indicators available for most countries, and indicators that are not currently produced or that have not yet been defined exactly at international level.

### The Link between SDGs and NRRP

After the scenario of the Covid-19 pandemic and the crisis that has overwhelmed all EU countries, Europe has met through the Next Generation EU (NGEU also called Recovery Fund), a 750-billion-euro plan, consisting of about half of the grants, agreed by the European Commission to support Member States in the post-pandemic recovery.

The main component of the NGEU program is the Recovery and Resilience Facility (RRF), which has a duration of six years, from 2021 to 2026, and a total size of €672.5 billion. The other component is the Recovery Assistance Package for Cohesion and Territories of Europe (REACT-EU).

The NRRP (National Recovery and Resilience Plan) is nothing more than the document required by the European Commission to each of the Member States to access the funds of the Recovery and Resilience Facility (RRF).

For Italy, the Plan is developed around three strategic axes shared at European level: digital transition and innovation, ecological transition, social inclusion and territorial rebalancing.

The Italian NRRP is divided into 6 missions, 16 components and 151 investments distributed as follows:

- Mission 1: Digitalization, innovation, competitiveness, culture and tourism
- Mission 2: Green Revolution and Ecological Transition
- Mission 3: Infrastructure for sustainable mobility
- Mission 4: Education and research
- Mission 5: Cohesion and inclusion
- Mission 6: Health

**Fig. 2:**
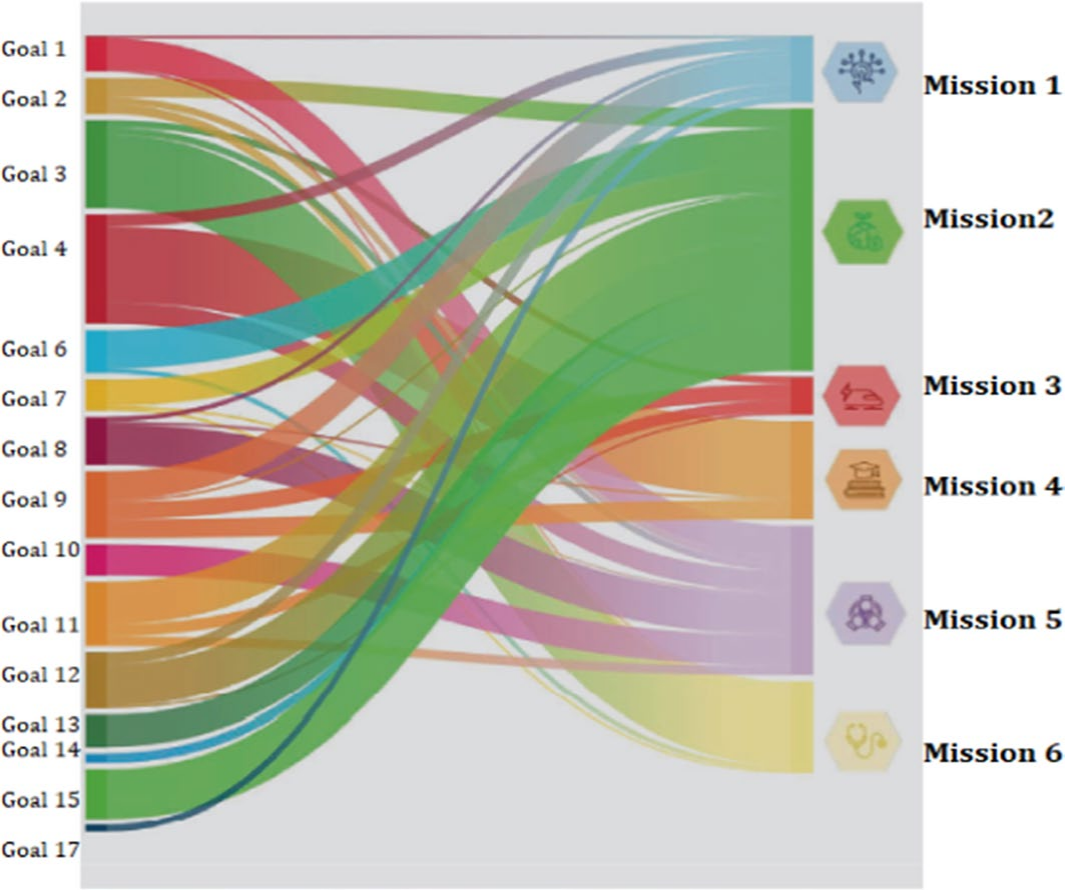
Relationships between SDGs indicators and NRRP missions

Sustainability indicators can be useful tools for monitoring the progress of the National Recovery and Resilience Plan. To this end, Istat proposes in the Report on the SDGs a mapping of the correspondences between the 17 Sustainable Development Goals and the 6 Missions envisaged by the NRRP (Fig. 2). This representation makes it possible to immediately define the SDGs indicators that can be useful tools for achieving the missions of the NRRP.

## A Multidimensional Approach for the Identification of the Areas that Require Nrrp Interventions

### The Fuzzy Approach

The fuzzy logic goes back to the initial work of Zadeh (1965), later taken up by Dubois and Prade (1980). The fuzzy theory is developed starting from the assumption that each unit is not uniquely associated with a single unit, but simultaneously with all the categories identified, on the basis of bonds of different intensity (degrees of association).

The first measure based on fuzzy set theory, called TF (Totally Fuzzy), was suggested by Cerioli and Zani (1990). Cheli and Lemmi (1995) have proposed a generalization of this approach, called Totally Fuzzy and Relative (TFR) which consists in defining the measure of the degree of belonging of an individual to the fuzzy totality, between 0 and 1.

Given a set ***X*** of elements, any blurred subset $x \in {\rm X}$ ***A*** of ***X*** is defined as follows:

1 $$ {\mathbf{A}} = \left\{ {{\mathbf{X}},f_{A} (x)} \right\} $$

where $f_{A} (x):{\mathbf{X}} \to \left[ {0,1} \right]$ is called the blurred subset membership *function ****A*** and indicates the degree of belonging of *x* to ***A*** where:

- if $f_{A} (x) = 0$ it occurs that *x* does not belong to **A*;***
- if $f_{A} (x) = 1$ it occurs that *x* belongs completely to ***A;***
- if $0 < f_{A} (x) < 1$ it occurs that *x* partially belongs to A, with a degree of belonging that is greater the closer it is to 1.

If we suppose to observe k indicators for each family, the function of belonging of the i-th family to the blurred subset can be defined as follows:

2 $$ f(x_{i.} ) = \frac{{\sum\nolimits_{j = 1}^{k} {g(x_{ij} ).w_{j} } }}{{\sum\nolimits_{j = 1}^{k} {w_{j} } }}i = 1, \ldots ,n $$

where g(x_*ij*_) measures the probability of membership of each unit and w_*j*_ derives from a weighting system, as given by generalizing the one proposed by Cerioli and Zani (1990):

3 $$ w_{j} = \ln \left[ {1/\overline{{g(x_{j} )}} } \right],\quad \quad \quad {\text{where}}\quad \overline{{g(x_{j} )}} = \frac{{\sum\nolimits_{i = 1}^{n} {g(x_{ij} )} }}{n}\quad \quad (j = 1,2,...,k). $$

When the average function $\overline{{g(x_{j} )}}$ the corresponding weight w_*j*_ is equal to zero, while when $\overline{{g(x_{j} )}}$=0 w_j_ is not defined, or rather X_*j*_ is not an appropriate indicator for that collective.

### The results of the application of Totally Fuzzy and Relative approach

Our work focused on the analysis of the indicators useful for Mission 2 (Green Revolution and Ecological Transition) of the National Recovery and Resilience Plan. It provides for investments and reforms to support the circular economy and the improvement of waste management. The Mission also includes interventions on territorial security, hydrogeological risk, protection of greenery and biodiversity, elimination of water and soil pollution, and availability of water resources.

The main components of this mission are:

-M2C1: Circular economy and sustainable agriculture.
- M2C2: Renewable energies, hydrogen, grid, and sustainable mobility.
- M2C3: Energy efficiency and building renovation.
- M2C4: Protection of the territory and water resources.

The analysis of Mission 2 (Green Revolution and Ecological Transition) finds ample space in the SDGs, creating important interconnections between the various indicators present in the individual Goals and the objectives of the Mission itself.

The indicators were chosen based on their relevance to the objectives of the mission and on the availability of data on a provincial basis update to 2020. We considered 4 SDGs indicators, selected from Goals 6, 7, 11, and 12, which may be of significant interest for the achievement of Mission 2. These indicators will then be attributed to the individual components of the mission (Table 1).

**Table 1:** Goal, indicators, measures e source of SDGs data

Goal	Indicators	Measures	Source of data
Goal 6.1.1:	Percentage of population benefiting from safely managed drinking water services	Irregularities in water distribution	(Istat, 2020, percentage values)
Goal 7.2.1:	Share of energy from renewable sources in total final energy consumption	Electricity from renewable sources	(Terna Spa, 2019, percentage values)
Goal 11.7.1:	Average percentage of the urbanized area of cities that is used as a public space, by gender, age and people with disabilities	Incidence of urban green areas on the urbanized surface of cities	(Istat, 2019, m^2^ per 100 m^2^ of urbanized area)
Goal 12.5.1:	National recycling rate, tons of recycled material	Separate waste collection	(Istat elaboration on Ispra data, 2019, percentage values)

Source: our elaboration on SDGs

For each main component we can use the following indicators:

- M2C1: Circular economy and sustainable agriculture:
  - Electricity from renewable sources
- M2C2: Renewable energy, hydrogen, grid, and sustainable mobility:
  - Separate waste collection
- M2C4: Protection of land and water resources:
  - Irregularities in water distribution
  - Incidence of urban green areas on the urbanized surface of cities

The Total Fuzzy and Relative method was applied to the data of all the Italian provinces. For this set of indicators was calculated: the minimum value, the maximum value, the average, and the fuzzy value (Table 2). Of particular interest is the analysis of the weights *w*_*i*_ which indicate the relevance of the indicator on the considered set: high values ​​denote a strongly discriminating condition of that index on the final fuzzy result. In our case, the values ​​of the weights of the highest indicators are related to the "share of urban green incidence on the urbanized surface of cities" (*w*_*i*_ = 0.93). On the other hand, the weights of the other indicators appear less discriminatory.

**Table 2:** Results of the application of the TFR method in relation to the distribution function and the weights of the different indices. Source: our elaboration on SDGs

Components	Indicators	Minimum	Maximum	Mean	Gmean	Weight *w*_*j*_
M2C1: Circular economy and sustainable agriculture	Separate waste collection	7.8	84.1	47.8	0.57	0.24
M2C2: Renewable energy, hydrogen, network, and sustainable mobility	Electricity from renewable sources	3.2	100	39.1	0.37	0.43
M2C4: Protection of land and water resources	Irregularities in water distribution	24.6	91.4	63.4	0.61	0.22
	Incidence of urban green areas on the urbanized surface of cities	0	100	20.5	0.12	0.93

**Table 3:** Composition in absolute values and percentages of provinces by membership of fuzzy classes. Source: our elaboration on SDGs

Fuzzy value	Number of provinces	%
0,0 ┤0,2	28	25
0,2 ┤0,4	48	44
0,4 ┤0,6	27	25
0,6 ┤0,8	4	4
0,8 ┤1,0	3	3
**Total**	**110**	**100**

Bold values indicate the total values

As a result of the application, we have classified the Italian provinces based on fuzzy values ​​(Table 3). Recall that high values ​​are significant of provincial situations in good health and therefore do not require national interventions, vice versa low values ​​are significant of situations of difficulty.

**Fig. 3:**
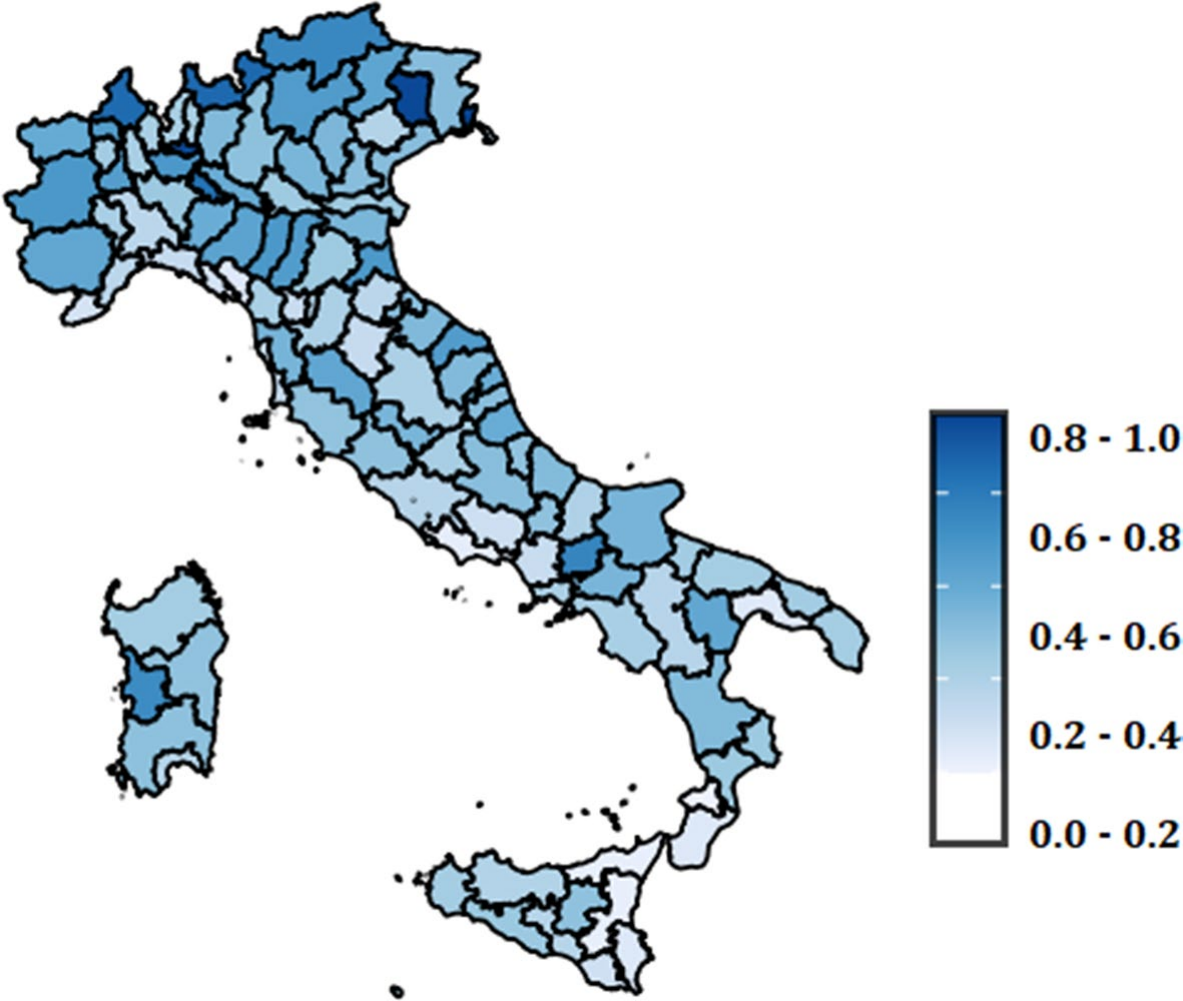
Territorial distribution by region of the fuzzy values for Mission 2 of the NRRP

Applying the Total Fuzzy and Relative method on the data of all Italian provinces, we obtain the following cartography (Fig. 3). It clearly emerges that there is no clear distinction of the provinces between Northern Italy and Southern Italy. The provinces with particularly high values close to 1 (Gorizia, Pordenone, and Monza) are mainly located in the North and are those that do not require large investments. On the contrary, those with values close to zero, denote critical situations and would require investment funds; in particular, we refer to the provinces of Calabria (Vibo Valentia, Reggio Calabria) and Sicily (Catania, Messina, Syracuse).

## DBSCAN Method

### Description of Algorithm

One of the main algorithms that allows to consider areas with higher density than others is the DBSCAN (Density Based Spatial Clustering of Application with Noise) (Ester et al., 1996). The key concept lies in the proximity of the models, the density of the neighboring points must exceed a certain threshold, on the contrary the density calculated in the so-called noise zones must not exceed this threshold.

The shape around the model depends on the type of distance function chosen for two pattern* p* and *q* indicated with *dist(p;q),* which is chosen according to the application considered. The algorithm needs two input parameters* ε* and *MinPts* to diversify the points in the data set into core points, border points, and noise points.

Some definitions are needed to formally describe the algorithm (as proposed by Ester et al., 1996 and reformulated in Montrone et al., 2011a, 2011b; 2012) of directly density-reachable, density-reachable, and density-connected.

- A point *p* is *directly density-reachable* from point *q* with respect to *ε* and *MinPts* in a set of points *D* if
- $$N_{\varepsilon } \left( q \right) = \left\{ {p \in D|dist\left( {p,q} \right) \le \varepsilon } \right\}$$.
- $\left| {N_{\varepsilon } \left( q \right)} \right| \ge MinPts$ (Means that *q* is a *core point*. Other points can be directly density-reachable only by core points)
- A point *p* is *density-reachable* from a point *q* with respect to *ε* and *MinPts* in the set of points *D* if there is a chain of points $$p_{1} , \ldots ,p_{n} $$ with $$p_{1} = q $$ and $$p_{n} = p, $$ such that $$p_{i} \in D$$ and $$ p_{i + 1}$$ is directly density-reachable from $$p_{i}$$ with respect to $$ \varepsilon$$ and *MinPts*.
- A point *p* is *density-connected* to point *q* with respect to *ε* and *MinPts* in the set of points *D* if there is a point $o \in D$ such that both *p* and *q* are density-reachable from *o* with respect to *ε* and *MinPts* in *D*.

A cluster understood as the highest density area is defined as a set of points density-connected that is maximum relative to density-reachability, and noise is the set of points not contained in any cluster.

The insertion of density-reachable points is iterated by inserting points directly density-reachable. If ${N}_{\varepsilon }\left(p\right),$ that is the *ε* -neighborhood of a point *p*, has more than MinPts points, a new C cluster is created. Then, the *ε* -neighborhood of all q points in C that have not yet been processed is checked, if ${N}_{\varepsilon }\left(q\right)$ contains multiple *MinPts* points, q neighbors that are not already contained in C are added to the cluster, and their *ε*-neighborhood is checked in the next step. When no new point can be added to the current C cluster, the procedure is stopped.

The advantages of DBSCAN are many, in fact it detects clusters of any size and shape; does not require a priori the number of clusters to be searched for and the input parameters with which to start the algorithm; automatically determines noise points. The algorithm requires only one distance function and two input parameters, the choice of which is very important. We will see in the application how to choose these input parameters.

### Application: Identification of Dense Areas with DBSCAN

The DBSCAN algorithm has been applied to the provinces to aggregate provinces even from different regions to find spatially close and contiguous areas with common characteristics to better organize territorial planning.

Based on the fuzzy value, the provinces were aggregated through the DBSCAN algorithm.

To define the optimal value of *ε* we are computing the k-nearest neighbor distances in a matrix of points. The idea is to calculate, the average of the distances of every point to its k nearest neighbors. The value of k is specified by us and corresponds to *MinPts.*

**Fig. 4:**
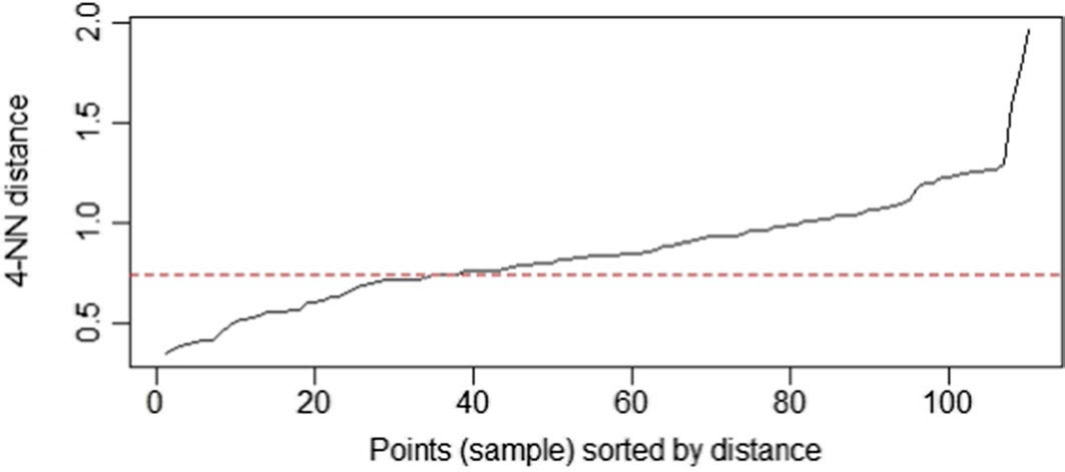
k-distances plot for optimal $\varepsilon$

Next, these k-distances are plotted in an ascending order. The aim is to determine the inflection point which corresponds to the optimal *ε* parameter (Fig. 4).

**Fig. 5:**
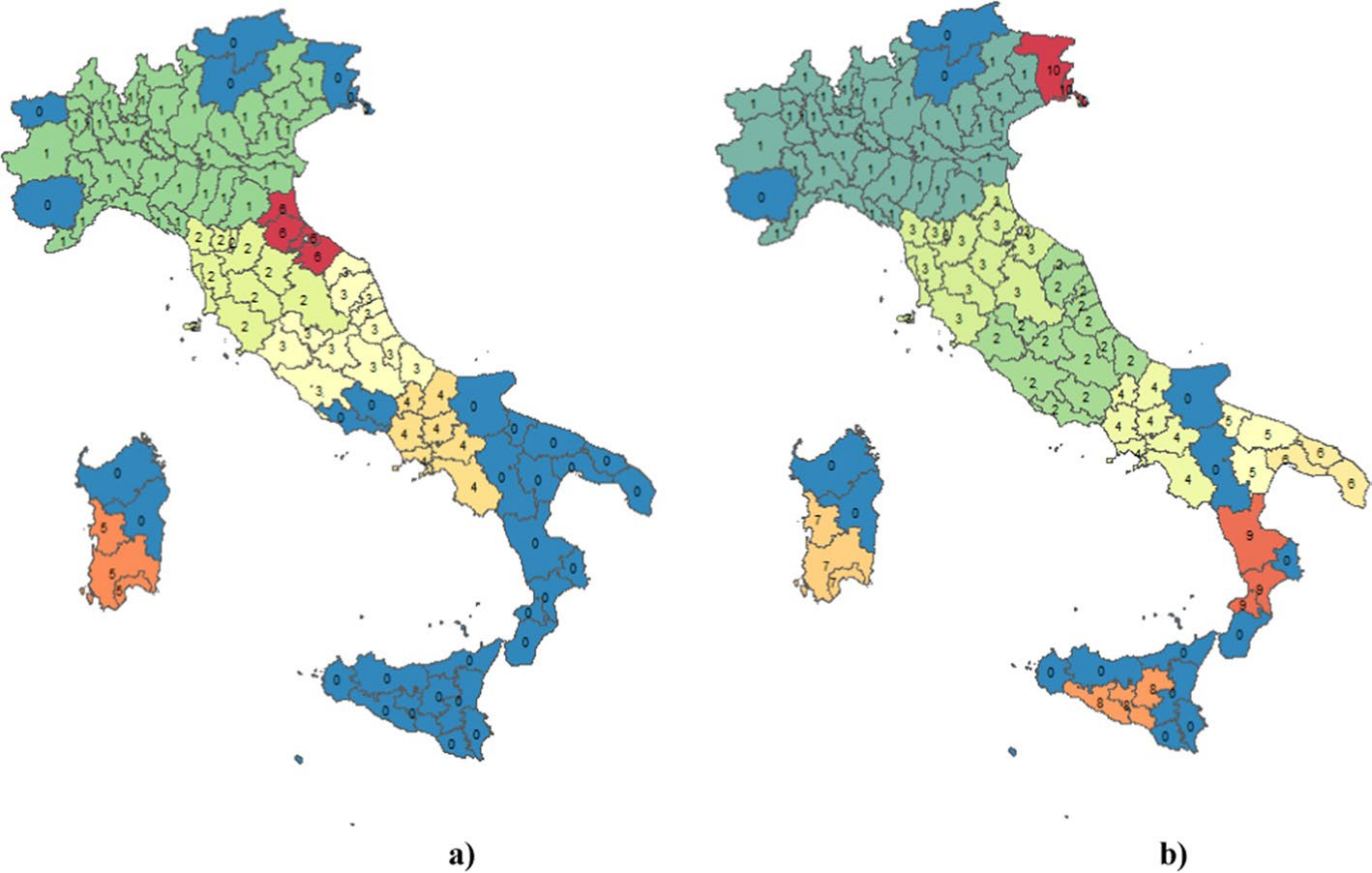
Identification of dense areas with DBSCAN ( $=0.75$ and *Minpts* = 3 in **a** and *Minpts* = 4 in **b**

The two cartographies are elaborated with the same *ε* value equal to 0,75, while *Minpts* has been varied from 3 to 4; for our application, we have empirically verified that the optimal value to be attributed to *Minpts* is 4, decreasing its value increases the number of clusters (Fig. 5). For the first cartography with $\varepsilon$ value equal to 0.75 and *Minpts* equal to 4, six clusters are obtained, in blue the noise points are colored and indicated with the value 0. The clusters with greater fuzzy value and spatially close are indicated with the numbers 1, 3, 5, 6. In the second cartography with $\varepsilon$ value equal to 0.75 and *Minpts* equal to 3, ten clusters are obtained. The clusters with higher fuzzy value and spatially close are indicated with the numbers 1, 7, 10.

The clusters that have a higher fuzzy average value than the others there is a greater attention to green revolution and ecological transition. These clusters include the northern regions: Lombardy Piedmont Veneto, Friuli, and Emilia Romagna. In every cartography, moreover, a cluster in Sardinia is detected because the data are from 2019 and are not aligned with the new reordering of the provinces in 2021; the subdivision into cartography is taken from the ISTAT provincial codes of 2022 and the fuzzy value is repeated in the same provinces that were previously merged.

**Fig. 6:**
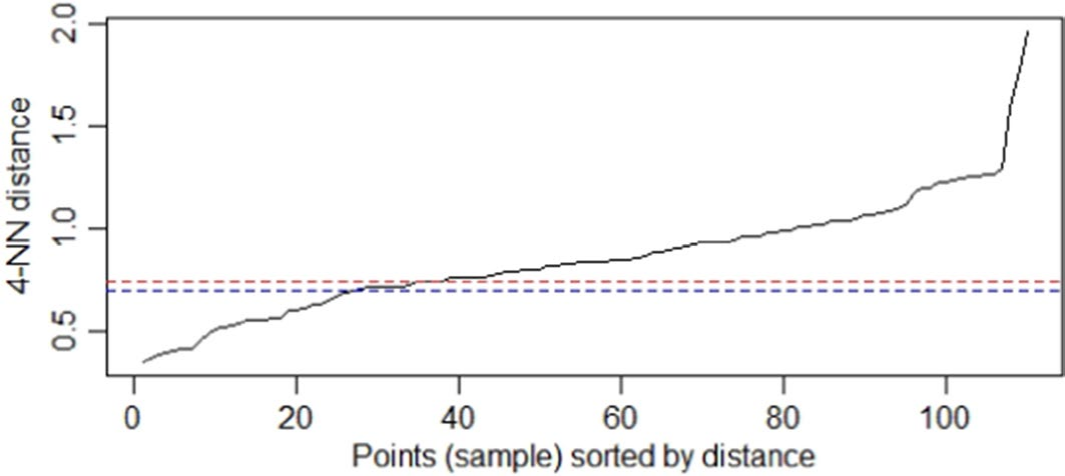
k-distances plot for $\varepsilon =0.70$ and $\varepsilon =0.75$

If we reduce the *ε* value and choose the *ε* value 0.70 (Fig. 6), the noise points increase and by increasing the value of *ε* the points are concentrated in a single cluster.

**Fig. 7:**
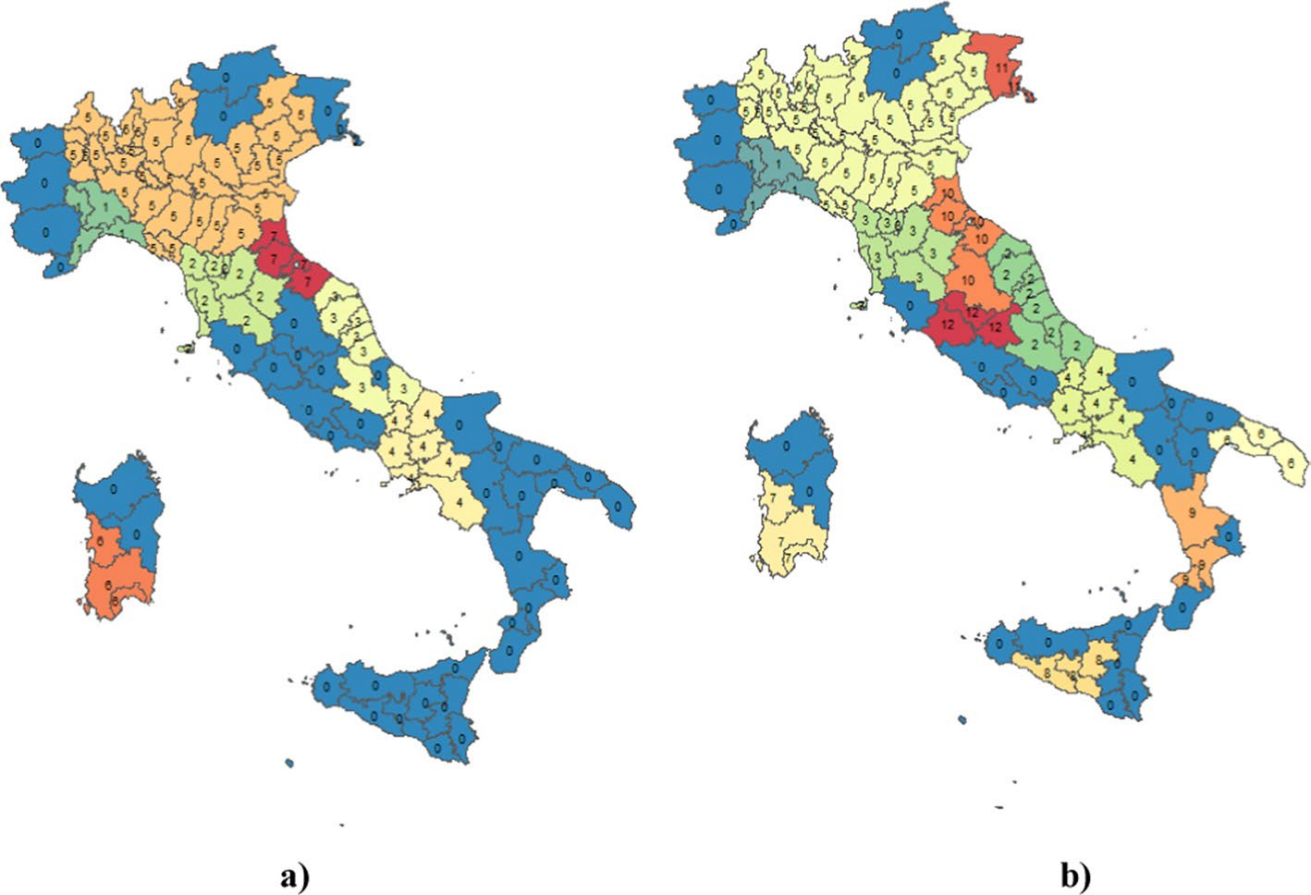
Identification of dense areas with DBSCAN ( $=0.70$ and *Minpts* = 3 in **a** and *Minpts* = 4 in **b**

In cartographies (Fig. 7), like in the previously example, at the same *ε* value equal to 0,70, *Minpts* has been varied from 3 to 4.

In Fig. 7a) with *ε* value equal to 0.70 and *Minpts* equal to 4, seven clusters are obtained in blue, the noise points are colored and indicated with the value 0. The clusters with greater fuzzy value and spatially close are indicated with the numbers 3, 5, 6, 7. In Fig. 7b) with *ε* value equal to 0.70 and *Minpts* equal to 3, twelve clusters are obtained. The clusters with higher fuzzy value and spatially close are indicated with the numbers 2,5,7, 11. With this value of *ε* (0.70) to the clusters identified previously is also added the cluster formed by the provinces of Marche and Abruzzo with a high average fuzzy value.

## Conclusions

The results of the fuzzy analysis developed in this work show that it is necessary to integrate the usual methods of distributing funds of NRRP with adequate tools for territorial rebalancing that, for each aspect, can support precisely the regions that start from the most disadvantaged situations. The DBSCAN algorithm shows several advantages: it detects clusters of arbitrary size and shape, it does not require in advance the number of clusters to be searched for and the initial patterns with which to start the algorithm, it automatically determines noise points, it is applicable to any metric space, it can follow the shape of the clusters and it requires only one distance function and two input parameters. The analysis of clusters using a density-based approach aims to identify areas where a given phenomenon assumes values significantly higher than those recorded in other parts of the territory.

Through the evaluation of the NRRP mission 2 and its components, territorial disparities among the Italian regions have been highlighted. According to the outcomes of this work, the territorial disparities do not reflect the classic gap between North and South, but the disparities are also scattered within the two classic Italian macro-areas (Viesti, 2021). These aspects should guide the way for distributing resources and investments, as the Italian regions currently do not start from the same conditions (ASviS, 2020).

The current criterion used in the competitive calls, for the implementation of the NRRP, could lead to more disparities among regions because what happens is that rich regions, with more possibilities to respond to competitive calls (because, for example, equipped with a larger, more competent, or better trained regional bureaucracy), are able to draw more resources.

In other countries than Italy, such as France and the United Kingdom, for example, when considering biodiversity enhancement policies, the most disadvantaged sub-national areas are encouraged by specific instruments (OECD, 2021, 2022), as well as it is happening in Ireland with the promotion of circular economy (UNESCO, 2017).

Data analysis allows to develop an integrated approach for the evaluation of the government policies of the territory (Cohen, 2017; Bond, 2011; Fitzgerald et al., 2012) and for monitor the progress of the subsequent intervention policies of the Italian Government.

In conclusion, public policies should be very careful to the different situations as the conditions for rebalancing different starting situations do not arise spontaneously, especially in the weaker areas (Gemenne & Rankovic, 2021). The results of this study about the different starting situations of the Italian regions, with respect to the sustainability objectives, should be used to better distribute the huge NRRP resources, also considering these imbalances.
